# Supplementary material for: MetaRibo-Seq measures translation in microbiomes
Source: Nat Commun. 2020 Jun 29;11:3268. doi: 10.1038/s41467-020-17081-z (PMC7324362; doi:10.1038/s41467-020-17081-z)
Supplement: Supplementary file 10 — Supplementary Data 7 [file 41467_2020_17081_MOESM10_ESM.zip › File2/Confidence_High_Taxonomy/82680_out.krona.html]

Javascript must be enabled to view this page.

members
magnitude
magnitudeUnassigned
count
unassigned
taxon
rank

82680\_out

102

2
102
superkingdom

phylum
1239
102

class
186801
102

186802
102
order

101
186803
family

841

SRS019910\_contig\_number\_15004SRS076804\_contig\_number\_16173SRS078242\_contig\_number\_contig-100\_44306.81927SRS104693\_contig\_number\_2215SRS147139\_contig\_number\_contig-100\_3266.35836
genus
5
101

species

SRS077335\_contig\_number\_2662
1
2049040


SRS011084\_contig\_number\_contig-100\_461.196498SRS011302\_contig\_number\_27069SRS011405\_contig\_number\_contig-100\_2017.98878SRS012849\_contig\_number\_35474SRS013951\_contig\_number\_9895SRS014412\_contig\_number\_20847SRS014459\_contig\_number\_contig-100\_904.223546SRS014923\_contig\_number\_17076SRS015065\_contig\_number\_17135SRS015431\_contig\_number\_42155SRS015578\_contig\_number\_30621SRS015663\_contig\_number\_36839SRS015890\_contig\_number\_24422SRS016095\_contig\_number\_20107SRS016335\_contig\_number\_42534SRS016954\_contig\_number\_25145SRS017433\_contig\_number\_14542SRS018984\_contig\_number\_25538SRS019068\_contig\_number\_18481SRS019161\_contig\_number\_contig-100\_840.233710SRS019381\_contig\_number\_14241SRS019397\_contig\_number\_10467SRS019445\_contig\_number\_26443SRS022137\_contig\_number\_11021SRS023346\_contig\_number\_6861SRS024331\_contig\_number\_7839SRS024492\_contig\_number\_10881SRS042628\_contig\_number\_36372SRS044535\_contig\_number\_contig-100\_6591.50981SRS045645\_contig\_number\_contig-100\_1469.142640SRS045739\_contig\_number\_contig-100\_1653.1653SRS048164\_contig\_number\_22893SRS048870\_contig\_number\_18492SRS049896\_contig\_number\_30802SRS049959\_contig\_number\_50972SRS050026\_contig\_number\_contig-100\_92.36431SRS050998\_contig\_number\_15183SRS051031\_contig\_number\_24756SRS051882\_contig\_number\_contig-100\_375.36803SRS052027\_contig\_number\_contig-100\_338.115735SRS058070\_contig\_number\_11143SRS058770\_contig\_number\_28839SRS063040\_contig\_number\_32070SRS064276\_contig\_number\_17942SRS064645\_contig\_number\_contig-100\_171.171SRS065504\_contig\_number\_7148SRS075021\_contig\_number\_6581SRS077194\_contig\_number\_1972SRS077454\_contig\_number\_15652SRS077730\_contig\_number\_489SRS097889\_contig\_number\_36638SRS097920\_contig\_number\_24625SRS097958\_contig\_number\_contig-100\_2896.101544SRS098827\_contig\_number\_contig-100\_279.83286SRS098881\_contig\_number\_contig-100\_89.169563SRS100021\_contig\_number\_366SRS101376\_contig\_number\_27185SRS103987\_contig\_number\_28323SRS104311\_contig\_number\_23735SRS104975\_contig\_number\_6697SRS105153\_contig\_number\_6852SRS1055022\_contig\_number\_contig-100\_5404.117929SRS140492\_contig\_number\_30349SRS142503\_contig\_number\_21287SRS142712\_contig\_number\_786SRS142890\_contig\_number\_9560SRS143070\_contig\_number\_contig-100\_1186.91091SRS143148\_contig\_number\_21034SRS143722\_contig\_number\_14205SRS143780\_contig\_number\_31838SRS144135\_contig\_number\_28297SRS144183\_contig\_number\_10212SRS144297\_contig\_number\_contig-100\_2099.92508SRS144362\_contig\_number\_31457SRS144506\_contig\_number\_21379SRS144714\_contig\_number\_27673SRS146764\_contig\_number\_31172SRS147271\_contig\_number\_37064SRS147346\_contig\_number\_11574SRS147766\_contig\_number\_contig-100\_2394.132314SRS147977\_contig\_number\_9450SRS148091\_contig\_number\_2925SRS148159\_contig\_number\_40219SRS148253\_contig\_number\_13557SRS148319\_contig\_number\_1316SRS148424\_contig\_number\_35776SRS148784\_contig\_number\_27535SRS148817\_contig\_number\_3163SRS149181\_contig\_number\_20535SRS893259\_contig\_number\_6366SRS893270\_contig\_number\_1260SRS893288\_contig\_number\_contig-100\_30.28988SRS893327\_contig\_number\_5252SRS893341\_contig\_number\_1970SRS893342\_contig\_number\_3352
95
360807
species

family
1
541000

genus
1
1263

species

SRS143417\_contig\_number\_20036
1
2293179
